# Silence‐Embedded Distress: A Case Report Conceptualizing a Culturally Mediated Trauma Phenomenon in Community‐Based Mental Health Practice in Rwanda

**DOI:** 10.1002/ccr3.73285

**Published:** 2026-08-06

**Authors:** Yves Gashugi

**Affiliations:** ^1^ University of Global Health Equity Burera Rwanda; ^2^ Rwanda Psychological Society Kigali Rwanda

**Keywords:** community‐based mental health, Rwanda, silence‐embedded distress, trauma

## Abstract

Silence‐Embedded Distress (SED) highlights how psychological hardships and trauma in community settings may remain unspoken and undetected by conventional diagnoses. Culturally sensitive, community‐based interventions can facilitate expression and recovery, emphasizing the importance of recognizing non‐verbal and relational manifestations of psychological suffering.

## 1 Introduction

*I am not crying because of what I have said, but because of what I wasn't able to say.*

Traumatic experiences in post‐conflict settings often manifest in ways that are not fully articulated [1, 2], particularly when cultural norms, collective history, and personal constraints shape emotional expression [3]. Silence is a complex and culturally mediated phenomenon that extends beyond the mere absence of speech [4]. In trauma‐affected contexts, it often functions as a protective mechanism, enabling individuals to regulate overwhelming emotions, navigate social expectations, or maintain personal and collective dignity [4]. Far from indicating resolution or emotional absence, silence can carry profound meaning, signaling unarticulated distress, suppressed memories, or relational tensions [2, 4].

Understanding silence as an expressive, rather than neutral, element of human experience is critical in trauma‐informed practice, as it allows practitioners to attend to what remains unspoken, reading between the lines of verbal and nonverbal communication [5]. Silence may function as a survival strategy, a protective mechanism, or a socially conditioned response [6], rather than a sign of healing [7].

Conventional mental health frameworks, which primarily focus on diagnosable conditions such as post‐traumatic stress disorder (PTSD), depression, or anxiety, may overlook subtler emotional states rooted in unarticulated trauma [8]. In Rwanda, survivors of the 1994 genocide against the Tutsi, during which over one million Tutsi were killed, and countless individuals subjected to physical, sexual, and psychological violence [9, 10] often carry internalized emotional burdens that remain unspoken yet profoundly affect their well‐being, interpersonal relationships, and daily functioning [11]. These long‐term psychological effects, including transgenerational trauma, manifest as pervasive fear, mistrust, and emotional distress [12].

Cultural norms that discourage open expressions of pain, frequently to avoid shame, ridicule, or social judgment, further reinforce silence, making it a commonly adopted coping mechanism in navigating personal and collective trauma [3, 13]. In clinical and community‐based practice, mental health practitioners encounter individuals whose suffering is substantial yet not fully expressed through conventional symptomatology.

### 1.1 Conceptual Emergence of Silence‐Embedded Distress (SED)

Observations from community‐based psychosocial support sessions, facilitated by a trained counselor (Umuhumurizamutima), revealed a recurring clinical pattern in which emotional distress manifested through tears, withdrawal, or behavioral hesitation without corresponding verbal disclosure of underlying experiences. This pattern is conceptualized in this case report as Silence‐Embedded Distress (SED).

Silence‐Embedded Distress (SED) refers to a culturally mediated form of psychological distress in which emotional suffering is consciously experienced but remains unexpressed due to perceived relational, cultural, or moral constraints. It is characterized by intact emotional awareness combined with sustained silence, maintained as a protective response to anticipated stigma, fear of judgment, or threats to identity, dignity, or social belonging.

Silence‐Embedded Distress (SED) is not proposed as a formal psychiatric diagnosis but rather as a descriptive clinical construct that emerged from community‐based mental health practice. It refers to a culturally mediated form of psychological suffering in which emotionally significant experiences remain unexpressed despite the individual's awareness of their emotional impact. In SED, silence functions as a protective response shaped by anticipated stigma, social judgment, relational concerns, or cultural expectations, resulting in distress that is carried internally and expressed indirectly through affective, relational, or behavioral manifestations. By emphasizing the role of culture, social context, and interpersonal meaning in shaping emotional expression, SED offers a framework for understanding forms of suffering that may not be fully captured through conventional symptom‐focused approaches. While the construct requires further empirical investigation and validation, it highlights the importance of attending to both spoken and unspoken dimensions of distress in trauma‐informed and culturally responsive mental health practice.

## 2 Case History

U.C. (initials used to preserve confidentiality) is a 25‐year‐old woman who participated in a community‐based psychosocial support program delivered through structured therapeutic sessions. She actively engaged in both the psychoeducational components and the facilitated community support groups known as *Urubuga Baho Neza* (Safe Spaces). Throughout the program, she consistently completed individual weekly exercises and participated meaningfully in group discussions.

### 2.1 Assessment Approach

Clinical assessment was conducted through continuous observation during group sessions, narrative inquiry, and individual supportive conversations facilitated by a trained mental health practitioner within the community‐based program. The focus of assessment was on emotional expression, interpersonal functioning, behavioral responses during structured exercises, and trauma‐related narrative content. No formal psychiatric diagnostic interview was administered; instead, a qualitative, context‐sensitive psychosocial assessment approach was used.

Table 1 presents the chronological progression of key clinical events observed throughout U.C.'s participation in the psychosocial support program. The timeline illustrates the emergence of acute emotional distress during the therapeutic intervention, subsequent disclosure of previously unspoken experiences, and the gradual improvement in emotional regulation and psychosocial functioning following culturally sensitive therapeutic support.

**TABLE 1:** Clinical timeline of key events in the case of U.C.

Phase/Session	Clinical events and observations
Sessions 1–6	Active participation in psychoeducational and group activities with no significant emotional disruption reported.
Sessions 7 (initial phase)	Introduction of the “empty chair” exercise during group intervention.
Sessions 7 (during intervention)	Acute emotional distress emerged, characterized by intense crying, withdrawal from group interaction, and emotional dysregulation.
Immediately after intervention	Gradual emotional stabilization followed by verbal disclosure of previously unspoken traumatic experience.
Subsequent sessions	Progressive improvement in emotional regulation, increased participation, and enhanced engagement in group activities.
Final session	Reported emotional relief, improved coping capacity, and readiness to reintegrate into community life and engage in peer‐support activities.

### 2.2 Mental Status Examination

During the acute emotional episode, U.C. appeared appropriately dressed with adequate personal hygiene. She was alert and oriented to person, place, and time. Speech was initially minimal due to emotional overwhelm but became coherent and goal‐directed after stabilization. Mood was described as intensely sad, with a congruent and labile affect. Thought processes remained logical and organized. There was no evidence of hallucinations, delusions, or formal thought disorder. Insight into her emotional experience was preserved, and judgment appeared intact. No suicidal or homicidal ideation was reported.

### 2.3 Therapeutic Process

The intervention included structured group‐based psychosocial support, psychoeducation, and experiential techniques, including the “empty chair” exercise designed to facilitate emotional expression and unresolved emotional processing. During the emotional episode, the therapist provided non‐intrusive containment, allowing emotional regulation without interruption.

Following stabilization, the participant engaged in individual supportive counseling sessions. These sessions focused on gradual emotional expression and narrative reconstruction of previously unspoken experiences. Over time, she transitioned from emotional suppression within group settings to increased verbal articulation and psychological processing of her experience.

### 2.4 Key Clinical Episode and Disclosure

During the “empty chair” exercise in Session 7, U.C. experienced acute emotional distress characterized by persistent crying and withdrawal from the group. After a period of silence and emotional stabilization, she verbalized her distress with the statement:

I am not crying because of what I have said, but because of what I was not able to say.

She subsequently disclosed a history of a past abortion, which she described as emotionally distressing and unresolved. She reported that her silence was maintained due to fear of social judgment, particularly concerns about stigma, relational rejection, and perceived loss of dignity within her sociocultural context.

### 2.5 Follow‐Up Information

In the weeks following the intervention, U.C. demonstrated progressive emotional stabilization. She reported reduced emotional overwhelm, improved affect regulation, and increased ability to participate in group and community‐based activities without withdrawal. At follow‐up, she expressed a sense of emotional relief and readiness to engage in peer‐support activities within her community.

### 2.6 Ethical Considerations

Written informed consent was obtained from the participant for publication of this case report and all accompanying anonymized clinical information. All identifying details were removed or modified to ensure confidentiality. The study was conducted in accordance with ethical principles for case reporting and community‐based mental health practice.

## 3 Differential Diagnosis

U.C.'s presentation was carefully assessed against established psychiatric and trauma‐related conditions. Despite intense emotional activation during the empty chair exercise, her distress was acute, situational, and relationally triggered rather than pervasive. She did not meet criteria for Major Depressive Disorder: there was no persistent low mood, anhedonia, sleep or appetite disturbances, psychomotor changes, cognitive impairment, or suicidal ideation. A PHQ‐9 screening would likely have scored below the diagnostic threshold [14]. Similarly, trauma‐related disorders such as PTSD or Complex PTSD were ruled out, as she exhibited no re‐experiencing, hyperarousal, dissociation, or generalized avoidance [15, 16]. Her anxiety was disclosure‐specific rather than generalized, making Social Anxiety Disorder unlikely [17].

The differential diagnosis further distinguished her condition from related psychological constructs. Emotional suppression was insufficient to describe the phenomenon, as U.C.'s unspoken narrative remained active and intensified over time [18]. Unlike repression, her silence was consciously maintained and socially mediated rather than unconsciously excluded [19]. Dissociation was absent, as she remained coherent, oriented, and emotionally connected [20]. Avoidance did not apply, since she remained aware of her internal experience but feared social consequences of disclosure [21]. Alexithymia was also not present, as she was capable of identifying and articulating her emotions once a safe context was provided [22]. Her affective response was not a manifestation of depression or trauma reliving, but a relationally embedded burden shaped by the social and cultural environment [23].

Taken together, these observations support conceptualizing her distress as Silence‐Embedded Distress (SED). SED is a culturally mediated, trauma‐informed expression of psychological suffering, characterized by relational and social embedding of unspoken emotional material. It arises at the intersection of personal trauma history, gendered expectations, cultural norms surrounding silence, anticipated stigma, and community relational structures. Crucially, the distress is maintained not solely by the original traumatic experience but by the weight of what cannot be safely expressed. Relief and symptom alleviation emerge only when safe and culturally sensitive spaces allow articulation of the so‐called previously suppressed experience [24]. SED is thus best understood as a trauma‐processing pattern and culturally shaped manifestation of distress, rather than a formal psychiatric disorder.

## 4 Results

Following the clinical intervention within the Urubuga Baho Neza (Safe Spaces) program, U.C. exhibited significant psychological and functional improvements over the course of engagement. At baseline, she presented with acute emotional dysregulation, withdrawal, and intense crying during the “empty chair” exercise, reflecting the psychological burden associated with Silence‐Embedded Distress (SED).

Through individualized therapeutic support and guided facilitation, U.C. gradually began to articulate previously unspoken experiences, including unresolved grief related to a past abortion and fears of social judgment. Within the first week of focused therapeutic engagement, she reported a noticeable reduction in emotional overwhelm, improved affect regulation, and the ability to participate actively in group exercises without intense withdrawal.

By the final session, U.C. demonstrated improved emotional stability, great engagement in group processes, and increased willingness to express herself within a supportive environment. She reported subjective relief following verbal disclosure of previously suppressed experiences and expressed readiness to continue her recovery process within her community setting, including interest in peer support involvement.

## 5 Discussion

The objective of this case report was to illuminate a clinically observed, culturally and socially mediated form of psychological distress identified in a single participant within a post‐conflict mental health setting in Rwanda, conceptualized as Silence‐Embedded Distress (SED). In this case, SED is understood as emerging at the intersection of an individual's trauma history, culturally shaped norms around emotional expression, gendered expectations, and perceived social consequences of disclosure. Evidence from collectivist societies indicates that trauma experiences are frequently shaped by sociocultural norms and social expectations, producing distress that does not align with conventional psychiatric categories [23, 25]. Such evidence is used here only to contextualize the present case rather than to infer population‐level patterns. Therefore, unspoken experiences in such contexts can manifest as significant emotional and relational burdens, even in the absence of diagnosable depression, PTSD, or anxiety disorders [8, 26].

Within this case, U.C. experienced an acute episode of intense crying, withdrawal, and affective overwhelm during a structured therapeutic intervention, triggered by the empty‐chair exercise. Notably, she did not meet diagnostic criteria for major depressive disorder, PTSD, or complex PTSD. She exhibited no persistent low mood, anhedonia, neurovegetative symptoms, intrusive memories, hyperarousal, avoidance, or dissociative features. Instead, her distress was relationally and culturally embedded, arising from the internal conflict between the desire to disclose a traumatic experience (a past abortion) and the fear of social judgment or stigma. Unlike emotional suppression, repression, or alexithymia, SED involves deliberate silence with full emotional awareness, maintained by culturally mediated social risk appraisal and communal relational structures. The intense affect observed during the intervention highlights the unresolved emotional burden that is temporarily held in silence until a safe and culturally attuned space allows expression [27, 28].

This case report is inherently limited by its single‐participant design, and therefore no generalization to other individuals or populations is intended. The observations presented should be interpreted strictly as preliminary, case‐based clinical insights. Future research should explore SED in larger and more diverse populations to assess cross‐cultural relevance. Although U.C. did not meet conventional diagnostic criteria for depression, PTSD, or complex PTSD, further empirical investigation is warranted to examine potential overlaps and distinctions with these disorders. SED occurred in a female participant, which may reflect how sociocultural expectations surrounding emotional expression can shape the way distress is experienced and disclosed within specific individual contexts [29].

## 6 Conclusion

Silence‐Embedded Distress (SED) represents a culturally mediated form of psychological suffering, characterized by the deliberate maintenance of silence in response to social, relational, and cultural constraints. This case report illustrates how SED manifests through affective and relational cues rather than conventional symptom clusters, underscoring the importance of culturally sensitive and trauma‐informed clinical approaches in community‐based mental health practice, which can enhance intervention effectiveness, facilitate emotional expression, and support recovery. Future research should examine SED across diverse populations to validate its conceptual framework and inform culturally responsive clinical guidelines.

## Author Contributions

**Yves Gashugi:** conceptualization, investigation, writing – original draft, writing – review and editing, visualization, validation, methodology, formal analysis, data curation, supervision, resources, project administration.

## Funding

The author has nothing to report.

## Ethics Statement

The patients signed a consent form prior to data collection and accepted the anonymous publication of the case study.

## Consent

Written informed consent was obtained to publish this report in accordance with the journal's patient consent policy.

## Conflicts of Interest

The author declares no conflicts of interest.

## Data Availability

The qualitative data supporting the findings of this study are retained by the first author for ethical and confidentiality reasons. De‐identified data may be made available upon reasonable request, subject to ethical approval and confidentiality considerations.
